# *Caenorhabditis elegans* as a Model to Assess the Potential Risk to Human Health Associated with the Use of Bisphenol A and Its Substitutes

**DOI:** 10.3390/ijms26052013

**Published:** 2025-02-25

**Authors:** Alžbeta Kaiglová, Zuzana Bárdyová, Patrícia Hockicková, Aneta Zvolenská, Kamila Melnikov, Soňa Kucharíková

**Affiliations:** Department of Laboratory Medicine, Faculty of Health Care and Social Work, Trnava University in Trnava, 918 43 Trnava, Slovakia; alzbeta.kaiglova@truni.sk (A.K.); zuzana.bardyova@truni.sk (Z.B.); patricia.hockickova@tvu.sk (P.H.); aneta.zvolenska@truni.sk (A.Z.); kamila.melnikov@truni.sk (K.M.)

**Keywords:** *Caenorhabditis elegans*, bisphenol A, bisphenol A analogs

## Abstract

Given its simplicity, *Caenorhabditis elegans* appears to be a promising model for future research on endocrine disruptors, including bisphenol A and its supposedly safer alternatives. The aim of this study was to investigate the impact of embryonic exposure of *C. elegans* to different concentrations (0.5, 1.0, and 5 µM) of bisphenol A and its analogs (bisphenol S, bisphenol F, and bisphenol AF) on selected biological characteristics of the nematode *C. elegans* and to compare them with an unexposed control group. Embryonal exposure of *C. elegans* to bisphenol A, as well as bisphenol S, F, and AF at concentrations of 0.5, 1.0, and 5 µM resulted in a significant influence on the percentage of hatched eggs and habituation to anterior stimuli (with significant results ranging from *p* ≤ 0.05 to *p* ≤ 0.001). The growth of *C. elegans* was also significantly impaired by bisphenol A, S, and AF in some concentrations (with *p*-values ranging from *p* ≤ 0.05 to *p* ≤ 0.001). Our findings confirm prior research that bisphenol A and its supposedly safer analogs exert a detrimental effect on diverse biological processes. Therefore, bisphenol A analogs should be employed with caution, particularly until a comprehensive risk assessment has been conducted.

## 1. Introduction

Sydney Brenner was the first to use the nematode *C. elegans* as a model organism. In 1974, he reported the results of his mapping of *C. elegans* mutations [1]. Since then, the importance and use of this nematode as a model organism have grown in many other scientific fields. The nematode offers numerous advantages, including economic and ethical benefits. The search for more acceptable alternatives has emerged from efforts to limit research on vertebrates. The model organism *C. elegans* can serve as an important milestone in elucidating the mechanism of action of various molecules and stimuli due to its ease of accessibility, cultivation, rapid life cycle, manipulation, and responses to various stimuli (change in behavior due to mechanical, chemical, osmotic, and thermal stimuli) [2,3,4,5].

Because of its simplicity, *C. elegans* is emerging as a promising model for future research on endocrine disruptors, which encompass a range of chemicals, including bisphenol A and its presumable safer alternatives employed in the manufacture of epoxy resins and polycarbonate plastics (critical components of numerous consumer goods, from food containers to water bottles) [6,7,8,9], along with many others. These compounds have the potential to disrupt the delicate hormonal balance and the normal endocrine system, thereby affecting the health and reproduction of animals and humans [10,11,12]. Molting, development, energy metabolism, reproduction, neural development, and several behaviors like feeding, defecating, and mating are all regulated by the endocrine system in *C. elegans* [13]. This nematode offers a valuable model organism to investigate the genetics and biochemistry of the endocrine system, providing crucial insights into signaling pathways relevant to human biology and medicine. Over the last decade, genetic analysis of worms has identified mutations in many genes that affect or are controlled by insulin signaling. A study conducted by Nagar et al. (2020) assessed the effects of endocrine disruptors on *C. elegans* through paraben exposure, finding that endocrine disrupting activity resulted in oxidative stress, altered vitellogenin gene expression, and adversely affected growth, behavior, and reproduction in worms [14]. Baumeister et al. have shown that insulin-like molecules, acting through the DAF-2 receptor and the DAF-16 transcription factor, influence an organism’s oxidative stress resistance and modulate metabolic pathways that impact lifespan [15]. Notably, *C. elegans* serves as a valuable biological model for identifying the effects of estrogen-like endocrine disruptor compounds and may offer crucial insights for evaluating the impact of persistent organic and environmental pollutants found in a multitude of everyday products [16,17].

Bisphenol A (BPA) is an organic, synthetic chemical that is utilized in the manufacturing of epoxy resins and polycarbonate plastics. It has been detected in a multitude of everyday products, including beverage and food packaging, plastic utensils, flame retardants, home electronics, thermal paper, dental sealants and composites, and other medical devices [18,19,20,21]. The environmental fate of BPA includes its release into the environment and subsequent distribution and accumulation in different ecosystems. The widespread use of BPA (a documented endocrine-disrupting chemical) has resulted in continuous exposure to humans, thereby posing a significant threat to human health. BPA’s hazardous properties and concerns over its effects on human health led to the development of BPA-free plastic for food packaging and storage containers. A BPA analog, such as BPS, BPF, or BPAF, is often incorporated into these materials to increase their hardness and durability. While these analogs were initially considered as safer alternatives, recent studies suggest that they may in fact exhibit similar endocrine-disrupting effects. Experimental findings have demonstrated a correlation between BPA analogs and the disruption of multiple biological processes, including reproductive disorders [22,23,24,25,26,27,28], cardiovascular disease [29], obesity [30,31], type 2 diabetes [32], neurobehavioral changes [33,34], and a possible increased risk of certain types of cancers [35].

While research has shown that BPA and its analogs can interfere with biological processes in living organisms in various ways, these compounds also pose a significant risk to aquatic ecosystems. For example, Moon et al. (2024) have addressed the monitoring of risks associated with the presence of BPAF in the aquatic environment [36]. The results of their research indicate that BPAF concentrations in some areas are reaching levels that are of significant concern to these ecosystems. In addition, these BPAFs can bioaccumulate, posing a potential risk to human health as well [36]. These facts highlight the necessity for comprehensive investigations into the safety of BPA and its analogs. Therefore, the objective of our study was to investigate the effect of different concentrations of bisphenol A (BPA) and its alternatives—bisphenol S (BPS), bisphenol F (BPF), and bisphenol AF (BPAF)—on selected biological characteristics (hatchability, habituation behavior, and growth) of the nematode *C. elegans* and to compare them with an unexposed control group. 

## 2. Results

### 2.1. The Effect of BPA and Its Analogs (BPS, BPF, BPAF) on the Hatchability of C. elegans

**Figure 1:**
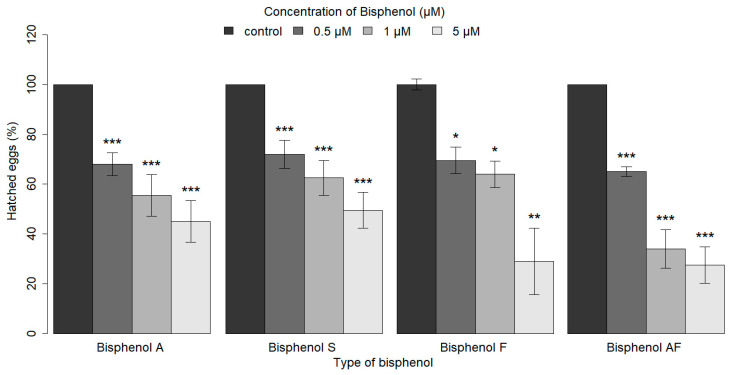
The effect of BPA and its analogs (BPS, BPF, BPAF) on the hatchability of *C. elegans*. A bar graph showing the median of the parameter of interest was used for graphical visualization, with error bars representing the standard error of the median (SEM). The error bars become invisible in groups exhibiting low variability, where the interquartile range (IQR) and the standard error of the mean (SEM) are both zero. Asterisks indicate statistically significant differences between groups (* *p* ≤ 0.05, ** *p* ≤ 0.01, *** *p* ≤ 0.001). As the concentrations of BPA and its analogs (BPS, BPF, and BPAF) increased, a significantly lower percentage of *C. elegans* eggs that hatched was observed (* *p* ≤ 0.05, ** *p* ≤ 0.01, *** *p* ≤ 0.001). The statistical significance of differences compared to the control group is indicated above the respective columns. The statistical analysis was performed using the Kruskal–Wallis test with post hoc analysis.

The changes in nematode hatchability were monitored 18 h after the exposure of eggs to bisphenols. The highest number of nematodes that hatched was observed in the control group. As the concentrations of BPA, BPS, BPF, and BPAF increased, a lower percentage of *C. elegans* eggs was observed to hatch. In our experiments, a significant reduction in hatchability was observed even at the lowest concentrations (0.5 µM) of bisphenols (BPA, BPS, BPF, and BPAF) tested (with significant results ranging from *p* ≤ 0.05 to *p* ≤ 0.001) (Figure 1). Furthermore, the hatchability of embryos exposed to BPA or BPA analogs (BPS, BPF, and BPAF) was not significantly different at any of the concentrations utilized, suggesting similar risk to health (Supplementary Figure S1).

### 2.2. The Impact of BPA and Its Analogs (BPS, BPF, BPAF) on the Habituation Behavior of Nematodes

The perception of mechanical stimuli induces alterations in the locomotion of *C. elegans* nematodes. Mechanosensory habituation is a form of non-associative learning that can be considered one of the simplest and most widespread forms of learning in nematodes and also in other animal species. The greatest advantage of habituation to an anterior touch stimulus is that the neural circuits in the head region that perform this behavior are very simple. They consist of only four bilateral sensory neurons, four interneurons, and a few motor neurons [37]. Nematodes respond to a gentle mechanical stimulus by moving backward, the so-called escape-and-return response. However, with repeated mechanical stimulation, these nematodes habituate to the stimuli and gradually decrease the distance of their escape response, i.e., backward retreat [38].

**Figure 2:**
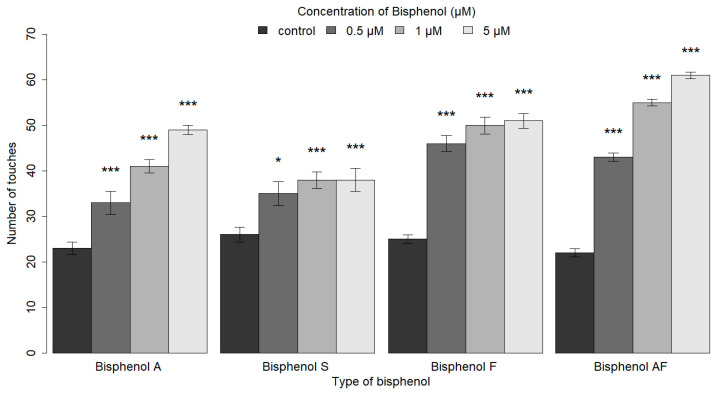
The effect of BPA and its analogs, including BPS, BPF, and BPAF, on the habituation behavior of nematodes. A bar graph was employed to illustrate the median of the parameter of interest, with error bars denoting the standard error of the median (SEM). The presence of asterisks indicates statistically significant differences between groups at the level of significance (* *p* ≤ 0.05, *** *p* ≤ 0.001). As with BPA, exposure to BPA analogs (in concentrations of 0.5, 1, and 5 μM) was found to significantly increase the number of stimuli required for habituation compared to the control group of animals (* *p* ≤ 0.05, *** *p* ≤ 0.001). The statistical significance compared to the control group is indicated above the respective columns. The statistical analysis was performed using the Kruskal–Wallis test with post hoc analysis.

The response to gentle anterior touch decreases with repeated stimulation. Gentle anterior touch stimulus was repeatedly given with a five-second interval between each stimulation until the animals stopped moving backward. It was observed that nematodes that had become habituated to the irrelevant stimuli no longer responded to them and began to move forward despite the persistent stimuli. As with BPA, exposure to BPA analogs (in concentrations of 0.5, 1, and 5 μM) resulted in a significant increase in the number of stimuli required for habituation compared to control animals exposed to S-Basal alone (with significant results ranging from *p* ≤ 0.05 to *p* ≤ 0.001 (Figure 2). A comparison of BPA and its analogs revealed a significant increase in the number of anterior touches required for habituation at concentrations of 0.5 μM and 1 μM for both BPF and BPAF, indicating that these BPA substitutes may pose an even more detrimental effect on behavior than BPA (Supplementary Figure S2).

### 2.3. The Effect of BPA and Its Analogs (BPS, BPF, BPAF) on the Growth of C. elegans

**Figure 3:**
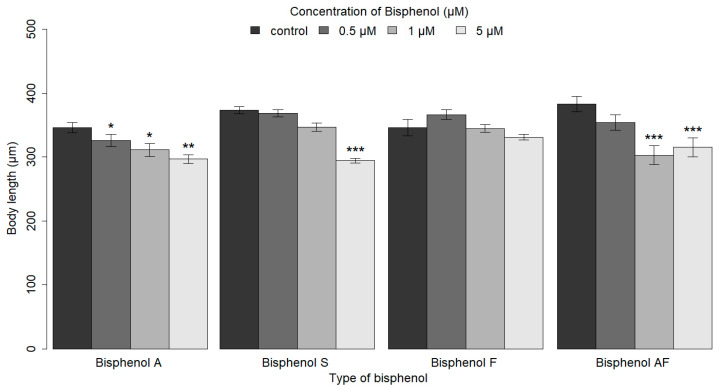
The impact of BPA and its analogs (BPS, BPF, and BPAF) on the growth of *C. elegans*. A bar graph demonstrating the median of the observed parameter with error bars indicating the standard error of the median (SEM). Asterisks indicate statistically significant differences between groups at the level of significance (* *p* ≤ 0.05, ** *p* ≤ 0.01, *** *p* ≤ 0.001). The body length of *C. elegans* nematodes was found to decrease significantly in response to increasing concentrations of BPA. Embryonic exposure to BPS at the highest concentration (5 µM) resulted in a statistically significant reduction in body length (*** *p* ≤ 0.001). Significant reductions in body length were observed for BPAF at concentrations of 1.0 and 5 μM (** *p* ≤ 0.01, *** *p* ≤ 0.001), whereas exposure to BPF had no significant effect on body length. The result shows a significant difference compared to the control group. To perform the statistical analysis, the Kruskal–Wallis test with post hoc analyses was employed.

To evaluate the impact of bisphenols on the growth of *C. elegans*, a control group was used to compare body length following exposure to varying concentrations (0.5, 1, and 5 μM) of BPA and its analogs (BPS, BPF, and BPAF). Our findings demonstrated that *C. elegans* nematodes in the L1 larval stage (18 h post-seeding at 20 °C) exhibited a significantly reduced body length when exposed to increasing concentrations of BPA (0.5, 1, and 5 μM) (*p* ≤ 0.01). Similarly, embryonic exposure to a concentration of 5 μM BPS resulted in a significant reduction in body length (*p* ≤ 0.001). Significant reductions in body length were observed in nematodes exposed to concentrations of 1 and 5 μM BPAF (*p* ≤ 0.05), while exposure to BPF had no significant effect on body length (Figure 3).

## 3. Discussion

Regulatory restrictions on BPA in some products due to health concerns have led to the replacement of this substance with its structural analogs. Even though these analogs are chemically different from BPA, little is known about their biological activity and potential health risks. The limited regulatory measures for these substances have resulted in their use in a wide variety of products [27,39,40]. This trend gives rise to concerns regarding the potential for the replacement of one problem with another, as it is likely that these analogs will also exhibit similar endocrine-disrupting effects. For this reason, increased attention is being paid to studies to identify the risks associated with exposure to BPA-free alternatives. A substantial number of studies have found that some BPA analogs can have a detrimental effect on the endocrine, reproductive, and nervous systems of living organisms. In the study conducted by Cabaton et al. (2009), for example, treatment of the HepG2 cell line with BPF resulted in induced oxidative stress and endocrine activity [41]. In the experiment of Ullah et al. (2018), male rats (22 days old) were exposed to BPA and its analogs (BPB, BPF, and BPS) in drinking water (at doses of 5, 25, and 50 μg/L) for 48 weeks. This research indicates that exposure to BPA and certain of its analogs (BPB, BPF, and BPS) alters spermatogenesis and induces oxidative stress in many tissues [42]. An in vitro study performed by Žalmanová et al. (2017) revealed that BPS adversely affects porcine oocyte maturation, even at levels lower than those to which humans are exposed daily. Following a 24 and 48 h in vitro culture period, cumulus-oocyte complexes exposed to varying concentrations of BPS (3, 30, 300 nM) demonstrated a significant dose-dependent decline in metaphase I and II stage achievement [27]. Furthermore, it has been documented that long-term exposure to low doses of BPS (0.001 ng.g/bw/day, 0.1 ng.g/bw/day, 10 ng.g/bw/day, and 100 ng.g/bw/day) has affected follicle development and oocyte quality in mice [26]. Regarding fecundity, Mersha et al. (2015) also demonstrated in experiments on the nematode *C. elegans* that embryos exposed to low doses of BPA (at a concentration of 1, 5, and 10 µM), as well as its analog BPS (at a concentration of 0.5, 1, 5, and 10 µM) for 4 h, laid significantly fewer eggs at maturity compared to unexposed controls [43]. This finding is consistent with our experimental results, which demonstrated a significant reduction in the hatching rate of *C. elegans* after exposure of embryos to the studied bisphenol compounds (BPA, BPS, BPF, BPAF) at the concentrations tested (0.5, 1, and 5 µM). In the course of early life stages, exposure to endocrine-disrupting chemicals, such as BPA, has been linked with the development of certain neurological disorders and the potential influence on social behavior [44]. It has been demonstrated that prenatal exposure to BPA is associated with differences in children’s brain microstructure, which may provide a biological rationale for the observed association between this exposure and behavioral symptoms in children [45]. However, it has recently been demonstrated that alternatives to BPA exhibit similar neurotoxicity and behavioral effects. Utilizing BPS, Kinch et al. (2015) reported that zebrafish embryos exhibited anxiety-like behaviors after undergoing neuronal proliferation [46]. Moreover, BPF, another BPA analog, has also been identified as a factor causing neuroinflammation in zebrafish embryonic development, affecting later cognitive abilities and brain tissue composition [47,48]. The majority of neurons in *C. elegans* exhibit a complex structure of connections with neighboring neurons and frequently express an unexpected assortment of signaling molecules. The complex neural system enables *C. elegans* to modify its behavior in accordance with habit and experience. Habituation refers to the steady drop in reaction to repeated stimuli [49]. The habituation of *C. elegans* to anterior touch stimuli after exposure to varying concentrations of BPA and its analog BPS has previously been observed. The results of these studies revealed that adult worms exposed to BPA and BPS during embryogenesis require more mechanosensory stimulation to habituate than unexposed worms [43,50]. In order to gain a more comprehensive understanding of the impact of bisphenols on the observed parameters, we extended our research by analyzing the effects of BPF and BPAF. The results obtained show that, similar to BPA and BPS, increasing concentrations of BPF and BPAF result in a substantial increase in the number of touches in comparison to the control. The findings of this study indicate that embryonic exposure of C. elegans nematodes to bisphenol A and its analogs (BPS, BPF, BPAF) may have a significant impact on their subsequent behavior, raising new questions about potential long-term consequences.

In general, a balance of thyroid hormones needs to be maintained to support normal growth. It is believed that BPA can affect the physiological functioning of several biological receptors, including the thyroid hormone receptor [51,52]. As demonstrated by Zhang et al. (2017), the potential for bisphenols, including BPA and some of its analogs (BPS, BPF), to disrupt the thyroid hormone signaling may result in a reduction in nematode body length following exposure [53]. This finding is partially consistent with the results of our study, which showed a significant decrease in body length after exposure to BPA and its analogs (BPS and BPAF). We found a slight reduction in nematode body length even after exposure to BPF, although this difference did not prove to be statistically significant. However, further research is necessary to ascertain whether this phenomenon occurs at a different developmental stage or after a longer exposure time. In the study by Wang et al. (2023), exposure to BPAF, or co-exposure to BPAF and nanoplastics, had a significant impact on other biological parameters (number of eggs laid, locomotion behavior of parental zebrafish, hatching rate), with the body length of zebrafish offspring significantly reduced compared to the control group [54]. In a similar vein, Xiao et al. (2019) documented that *C. elegans* exposed to 0.01 μM BPS exhibited a significant inhibition in growth, locomotion behavior, and impaired reproductive and antioxidant systems [55]. Subsequent experiments using the nematode *C. elegans* displayed that exposure to a concentration of 1 mM BPA led to significant abnormalities in development and growth. In addition to developmental delays and reduced body growth, reduced reproductive capacity and morphological changes to the tissues have also been observed. Similar negative effects, albeit in varying degrees, were also observed with exposure to BPA analogs (BPS, BPF, and tetramethyl bisphenol F [56].

The results of the present study are consistent with those of previous studies, which have shown the negative impact of bisphenols on a variety of biological processes. In accordance with the previous studies, the present findings indicate that exposure to BPA and its analogs can decrease reproductive capacity, impair locomotion activities, and alter body parameters in models, including *C. elegans*. Therefore, BPA analogs should be used with caution until a comprehensive and efficacious risk assessment has been conducted.

## 4. Conclusions

The findings of the present study indicate the potential of adverse behavioral and reproductive consequences associated with the utilization of bisphenol substitutes, namely BPS, BPF, and BPAF, despite their categorization as safer alternatives. In particular, we observed that embryonic exposure to BPA and its analogs, BPS, BPF, and BPAF, exerted significant effects on several developmental and behavioral outcomes. The percentage of hatched eggs significantly decreased, thereby indicating the potential for adverse effects on reproduction. Furthermore, these bisphenols interfered with habituation to anterior stimuli, suggesting possible disruptions in neurological function. Finally, an effect on *C. elegans* growth was observed, demonstrating that these substitutes may interfere with fundamental developmental processes. In view of these combined results, we are concerned about BPS, BPF, and BPAF’s presumed safety and urge further research into their effects on both development and behavior.

## 5. Materials and Methods

### 5.1. The Preparation of Nematode Growth Media (NGM) Plates

*C. elegans* nematodes were cultured on Nematode Growth Medium (NGM) plates seeded with *Escherichia coli* OP50. *E. coli* OP50, a uracil auxotroph, was used as a food source due to its restricted growth on NGM, which enabled more convenient observation of the nematodes. NGM plates were prepared according to the protocol of Merrow and Olmeda [57]. The stock solution was prepared by mixing 3 g NaCl, 17 g agar, 2.5 g peptone, and 975 mL distilled water. After autoclaving and cooling to 55 °C in a water bath, the following sterile solutions were added in an aseptic manner: 1 mL of 1 M CaCl_2_, 1 mL of cholesterol dissolved in ethanol (5 mg/mL), 1 mL of 1 M MgSO_4_ and 25 mL of 1 M KPO_4_. The prepared solution was then poured into sterile Petri dishes and left to solidify at room temperature in a laminar flow cabinet.

### 5.2. Cultivation of Escherichia coli OP50

The uracil auxotroph *E. coli* strain OP50, whose growth is restricted on NGM plates, was incubated for 24 h at 37 °C in Luria–Bertani (LB) liquid culture medium. As this facilitates better observation of worms, a limited bacterial lawn is preferred. Following the incubation period, the turbidity of the culture (*E. coli* OP50 in LB liquid culture medium) was evaluated visually, which corresponded to approximately 0.5 McFarland units. Subsequently, a 50 µL aliquot of the *E. coli* OP50 bacterial suspension was inoculated onto the surface of the NGM plates and left to soak. The prepared plates were stored at 4 °C until use.

### 5.3. Egg Harvesting

The M9 solution was pipetted onto the NGM plate with a sufficient number of eggs and adult gravid nematodes (i.e., approximately 72 h after transferring *C. elegans* from one Petri dish to another by “chunking”) and gently spatulated over the surface. The mixture was aspirated and transferred to a centrifuge tube. This step was repeated once more for maximum recovery. After centrifugation (1 min, 1400 RCF), the supernatant was removed, and a 25% solution of freshly prepared sodium hypochlorite solution (8.25 mL of sterile distilled water, 3.75 mL of 1 M NaOH, and 3 mL of bleach) was added to the pellet for 5 min. After brief mixing and vortexing, the pellet was centrifuged again, and the supernatant was aspirated as quickly as possible to avoid unnecessarily prolonged exposure to the sodium hypochlorite solution, which could also damage the eggs with prolonged exposure. Subsequently, the pellet was washed three times with M9 solution to remove residual sodium hypochlorite and dead nematodes, leaving only the nematode eggs.

### 5.4. Preparation of Bisphenol Solutions

High-purity BPA (4,4′-isopropylidenediphenol, 97%) and BPS (4,4′-sulfonyldiphenol, 99.7%) from Acros Organics (a division of Thermo Fisher Scientific Geel, Belgium), as well as BPF (4,4′-methylenediphenol, 98.0%) and BPAF (4,4′-hexafluoroisopropylidene-diphenol, 99%) from Sigma-Aldrich Intl GmbH, Buchs SG, Switzerland, were used for the experiments. Stock solutions of these bisphenols with a concentration of 100 μM were prepared in a 10% ethanol solution. From these stock solutions, working solutions in S-buffer (129 mL of 0.05 M K_2_HPO_4_, 871 mL of 0.05 M KH_2_PO_4_, and 5.85 g of NaCl) with final concentrations of 0.5, 1, and 5 μM were subsequently prepared according to the protocol previously described [50]. Due to the poor solubility of bisphenols, an ultrasonic homogenizer (sonicator) was used to accelerate their dissolution. The stock solutions were sonicated for 15 min at 37 °C.

### 5.5. Monitoring the Effect of Exposure of C. elegans Embryos to bisphenol A, S, F, and AF on Hatching Compared to Control Worms

*C. elegans* embryos were exposed to individual concentrations of BPA, S, F, and AF at 20 °C for 4 h and maintained on a Titramax 1000 shaker under constant agitation. At the end of the exposure period, the centrifugation microtubes were centrifuged at 1400 RCF for 30 sec. followed by washing with S basal solution to get rid of as much bisphenol as possible. The washing and centrifugation procedures were repeated 3 times to avoid unnecessary bias caused by inappropriately long exposure of nematodes to bisphenols. After the last centrifugation, the resulting supernatant was carefully aspirated so that approximately 100 μL of suspension of S-basal and embryos remained in the microtube. After thorough mixing, 25 μL of the suspension was plated on a Petri dish with NGM agar (seeded with the *E. coli* OP50 strain), and nematode embryos (eggs) were counted. Control worms that were exposed to the S-buffer alone underwent the same treatment procedure. The plates were incubated at 20 °C for 18 h. Using a stereomicroscope, the number of hatched nematodes in each plate was recorded.

### 5.6. Habituation Behavior of C. elegans

Following 48 h of development, *C. elegans* previously exposed to bisphenol concentrations, as well as controls, were assessed for habituation behavior, as outlined in previous studies [50,58]. Habituation to forward touch stimuli was performed using a glass stick with an adhered artificial eyelash, which was an ideal choice in terms of the required softness of touch. Nematodes were touched at approximately the same location in the head region at 5 s intervals. It was observed that nematodes that had become habituated to the irrelevant stimuli stopped responding to them (i.e., began to move forward despite the persistent stimuli). The number of touches required for habituation was recorded and evaluated.

### 5.7. C. elegans Larvae Body Length Measurement

Using a prep needle, *C. elegans* larvae (incubated at 20 °C for 18 h) were placed on microscope slides. Animal sacrifices were the result of heating the microscope slides over a flame. In addition, the heat-killed worms had their bodies straightened. A NIKON Eclipse 200 microscope coupled to a computer running metric measurement software was used to analyze the body length of heat-killed *C. elegans*.

### 5.8. Statistical Analysis

Statistical analysis was performed using the statistical software R-Project version 4.3.3. The significance between groups was assessed using the Kruskal–Wallis test. In the event of a significant result, a post hoc analysis was performed using the Pairwise–Wilcox test to identify specific differences between groups. The Shapiro–Wilk test was used to test the normality of the data distribution. The results were considered statistically significant at *p* < 0.05.

### 5.9. Ethical Approval

Although strict ethical approval is not required for experiments with *C. elegans*, the researchers in this study adhered to ethical principles and ensured that their research was conducted responsibly and humanely.

## Data Availability

The data analyzed in this current study are included in this manuscript and in Supplementary Materials. Additional analyses are available from the corresponding author upon reasonable request.

## Supplementary Materials

The following supporting information can be downloaded at: https://www.mdpi.com/article/10.3390/ijms26052013/s1.
