# Supplementary material for: Caenorhabditis elegans as a Model to Assess the Potential Risk to Human Health Associated with the Use of Bisphenol A and Its Substitutes
Source: Int J Mol Sci. 2025 Feb 25;26(5):2013. doi: 10.3390/ijms26052013 (PMC11900329; doi:10.3390/ijms26052013)
Supplement: Supplementary file 1 [file ijms-26-02013-s001.zip › ijms-3411690-supplementary.pdf]

International Journal of *Molecular Sciences*

MDPI

Supplementary Materials

Caenorhabditis elegans as a Model to Assess the Potential Risk to Human Health Associated with the Use of Bisphenol a and Its Substitutes

Alžbeta Kaiglová, Zuzana Bárdyová, Patrícia Hockicková, Aneta Zvolenská, Kamila Melnikov and Soňa Kuchariková *

Department of Laboratory Medicine, Faculty of Health Care and Social Work, Trnava University in Trnava, 918 43 Trnava, Slovakia; alzbeta.kaiglova@truni.sk (A.K.); zuzana.bardyova@truni.sk (Z.B.); patricia.hockickova@tvu.sk (P.H.); aneta.zvolenska@truni.sk (A.Z.); kamila.melnikov@truni.sk (K.M.)

* Correspondence: sona.kucharikova@truni.sk

Supplementary Materials

Supplementary Figure S1.
Supplementary Figure S2.

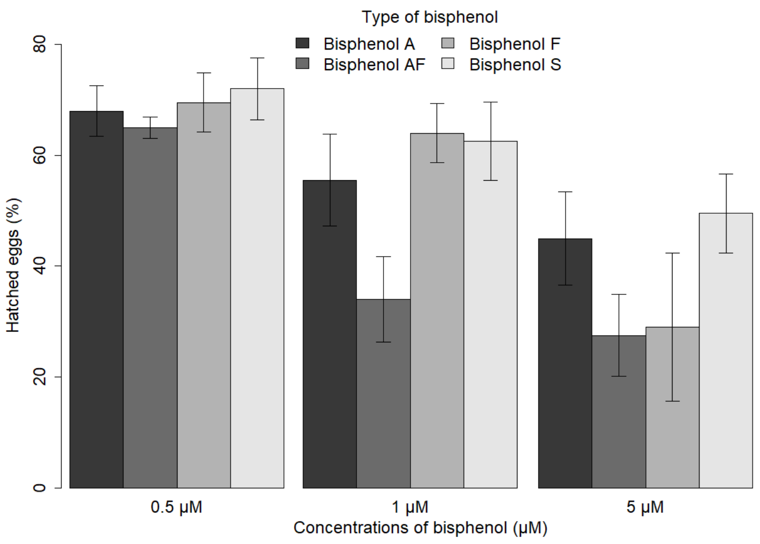

Figure S1: A comparison of the percentage of eggs that hatched after exposure of *C. elegans* embryos to BPA and its analogs (BPS, BPF, BPAF). A bar graph demonstrating the median of the observed parameter with error bars indicating the standard error of the median (SEM). The hatchability of embryos exposed to BPA or BPA analogs (BPS, BPF and BPAF) was not significantly different (at any of the concentrations used), suggesting similar risk to health. To perform the statistical analysis, the Kruskal–Wallis test with post hoc analysis were used employed.

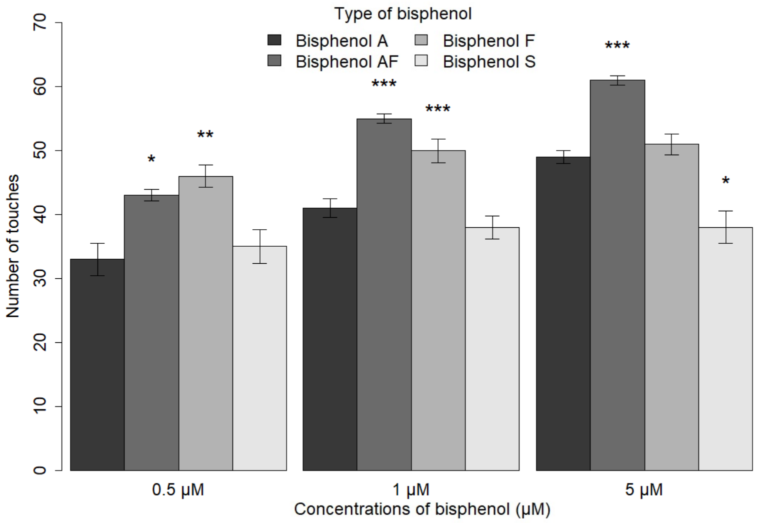

Figure S2: BPA and its analogs (BPS, BPF, BPAF): comparison of anterior touches required for habituation *C. elegans* after embryonal exposure. A bar graph was employed to illustrate the median of the parameter of interest, with error bars denoting the standard error of the median (SEM). The presence of asterisks indicates statistically significant differences between groups at the level of significance (*$p \leq 0.05$, **$p \leq 0.01$, ***$p \leq 0.001$). Comparison of BPA and its analogues showed that the number of anterior touches required for habituation was significantly increased after embyonal exposure to both BPF and BPAF at concentrations of 0.5, 1, and 5 μM in comparison to embryonal exposure of the same doses of BPA. This indicates that these BPA analogs may pose an even more detrimental effect on behavior than BPA. The statistical analysis was performed using the Kruskal–Wallis test with post hoc analysis.
